# Fatal Yellow Fever in Travelers to Brazil, 2018

**DOI:** 10.15585/mmwr.mm6711e1

**Published:** 2018-03-23

**Authors:** Davidson H. Hamer, Kristina Angelo, Eric Caumes, Perry J.J. van Genderen, Simin A. Florescu, Corneliu P. Popescu, Cecilia Perret, Angela McBride, Anna Checkley, Jenny Ryan, Martin Cetron, Patricia Schlagenhauf

**Affiliations:** ^1^Department of Global Health, Boston University School of Public Health, Massachusetts; ^2^Section of Infectious Diseases, Department of Medicine, Boston Medical Center, Massachusetts, USA; ^3^Travelers’ Health Branch, Division of Global Migration and Quarantine, CDC; ^4^Department of Infectious and Tropical Diseases, Groupe Hospitalier Pitié-Salpêtrière, Paris Sorbonne University, Paris, France; ^5^Harbour Hospital, Rotterdam, Netherlands; ^6^Carol Davila University of Medicine and Pharmacy, Victor Babes Clinical Hospital of Infectious and Tropical Diseases, Bucharest, Romania; ^7^Pontificia Universidad Catolica de Chile School of Medicine, Santiago, Chile; ^8^Hospital for Tropical Diseases, University College London Hospitals, London, United Kingdom; ^9^Royal Free Hospital, London, United Kingdom; ^10^Office of the Director, Division of Global Migration and Quarantine, CDC; ^11^World Health Organization Collaborating Centre for Travellers’ Health, Epidemiology, Biostatistics, and Prevention Institute, University of Zürich, Switzerland.

Yellow fever virus is a mosquito-borne flavivirus that causes yellow fever, an acute infectious disease that occurs in South America and sub-Saharan Africa. Most patients with yellow fever are asymptomatic, but among the 15% who develop severe illness, the case fatality rate is 20%–60%. Effective live-attenuated virus vaccines are available that protect against yellow fever (*1*). An outbreak of yellow fever began in Brazil in December 2016; since July 2017, cases in both humans and nonhuman primates have been reported from the states of São Paulo, Minas Gerais, and Rio de Janeiro, including cases occurring near large urban centers in these states (*2*). On January 16, 2018, the World Health Organization updated yellow fever vaccination recommendations for Brazil to include all persons traveling to or living in Espírito Santo, São Paulo, and Rio de Janeiro states, and certain cities in Bahia state, in addition to areas where vaccination had been recommended before the recent outbreak (*3*). Since January 2018, 10 travel-related cases of yellow fever, including four deaths, have been reported in international travelers returning from Brazil. None of the 10 travelers had received yellow fever vaccination. 

Five of the 10 cases were reported by ProMED since January 15, including two from Argentina and three from Chile; two of the travelers from Chile died. In addition, during January 1–March 15, 2018, five confirmed cases of yellow fever in unvaccinated travelers returning from Brazil were reported by GeoSentinel (http://www.istm.org/geosentinel), the global clinician-based sentinel surveillance system for travel-related illness among international travelers and migrants (*4*). These five yellow fever cases represent the first such cases identified by GeoSentinel (Table), which was initiated in 1995 by the International Society of Travel Medicine with support from CDC and now consists of 70 specialized travel and tropical medicine clinical sites around the world. The first of the GeoSentinel-reported cases occurred in a Dutch man aged 46 years who traveled to São Paulo state for 3 weeks during December 2017–January 2018. The second case occurred in a French woman, aged 42 years, who traveled to Minas Gerais state in Brazil for 4 weeks during December 2017–January 2018. She received a diagnosis of yellow fever in Brazil and was examined at a GeoSentinel site after returning to France to convalesce. The third and fourth cases occurred in a Romanian man, aged 34 years, and a Swiss man, aged 44 years, each of whom visited Brazil for approximately 2 weeks in February 2018. The fifth case was in a German man, aged 33 years, who spent a week in Brazil in late February. The Swiss and German travelers died from their illness (Table).

**TABLE:** Characteristics of five travelers to Brazil with yellow fever reported by GeoSentinel sites, January–March 2018*

Characteristic	Patient 1 (man)	Patient 2 (woman)	Patient 3 (man)	Patient 4 (man)	Patient 5 (man)
**Age (yrs)**	46	42	34	44	33
**Nationality**	Dutch	French	Romanian	Swiss	German
**Reporting site**	Netherlands	France	Romania	Switzerland	United Kingdom
**Area (state) of presumed yellow fever acquisition**	Mairiporã (São Paulo)	(Minas Gerais)	Ilha Grande (Rio de Janeiro)	Ilha Grande (Rio de Janeiro)	Ilha Grande (Rio de Janeiro)
**Signs/Symptoms**	Fever, headache, myalgia, nausea, vomiting, diarrhea	Fever	Fever, rash, myalgia, encephalopathy	Fever, petechial rash, arthralgia, vomiting, diarrhea	Fever, malaise, nausea, jaundice, hepatomegaly
**Clinical/Laboratory findings**	Hepatitis	Hepatitis, thrombocytopenia, neutropenia	Renal and hepatic failure	Renal and hepatic failure	Thrombocytopenia, renal and hepatic failure
**Yellow fever diagnostic testing**	Positive RT-PCR for YFV (urine, whole blood, plasma)	Positive RT-PCR (blood); positive IgM (initial diagnosis made in Brazil)	Positive PCR (serum, urine); YF IgM positive; IgG titers rising days 4–8	Positive PCR (blood)	Positive RT-PCR (serum, urine)
**Yellow fever vaccination status**	No	No	No	No	No
**Outcome**	Recovered	Recovered	Condition improving as of March 15, 2018	Died	Died

**Abbreviations**: IgG = Immunoglobulin G; IgM = Immunoglobulin M; PCR = polymerase chain reaction; RT-PCR = reverse transcription–PCR; YF = yellow fever; YFV = YF virus.

* In addition to the five patients reported by GeoSentinel sites, five additional cases of yellow fever have been reported by ProMED among persons who traveled to Brazil from Argentina (two) and Chile (three) since January 2018. Two of the patients from Chile died.

Among the 10 international travelers reported with yellow fever acquired in Brazil, eight acquired the disease on Ilha Grande, a forested island off the Rio de Janeiro coast, where one human and one nonhuman primate yellow fever case were reported in early February 2018 (*5*); of the eight patients who acquired the disease on Ilha Grande, four died. Another travel-related case of yellow fever was reported recently outside of Brazil (*6*).

Yellow fever is a potentially fatal illness that is preventable by vaccination. Yellow fever vaccination is recommended for all eligible persons aged ≥9 months, traveling to many areas in Brazil, including the states of São Paulo and Rio de Janeiro (especially Ilha Grande). Unvaccinated travelers should avoid traveling to areas where vaccination is recommended (https://wwwnc.cdc.gov/travel/notices). Travelers planning to visit areas in Brazil or elsewhere where yellow fever transmission is occurring should receive yellow fever vaccine at least 10 days before travel and follow recommendations for avoiding mosquito bites (https://www.cdc.gov/yellowfever/prevention/index.html). The Food and Drug Administration–approved yellow fever vaccine, YF-VAX, is currently unavailable in the United States because of manufacturing difficulties (*7*). An alternative yellow fever vaccine, Stamaril, is available through a limited number of U.S. yellow fever vaccination clinics. U.S. travelers should therefore plan ahead to obtain Stamaril because it might take more time to access one of these clinics. Clinicians assessing returned travelers should be aware of yellow fever signs and symptoms and maintain vigilance regarding the possibility of yellow fever exposure in travelers returning from Brazil or other areas with ongoing transmission of yellow fever.
